# A case of splenic abscess complicated with intra-abdominal free gas misdiagnosed as gastrointestinal perforation

**DOI:** 10.1093/jscr/rjag428

**Published:** 2026-06-03

**Authors:** Wenlong Du

**Affiliations:** Department of Anorectal Surgery, The People's Hospital of Linxia Hui Autonomous Prefecture, Linxia, China

**Keywords:** splenic abscess, gastrointestinal perforation, acute abdomen

## Abstract

The authors present a case of splenic abscess accompanied by intra-abdominal free gas, which was initially misdiagnosed as gastrointestinal perforation. This patient presented with acute onset, severe symptoms, and marked abdominal tenderness. Imaging studies, including chest X-ray and abdominal computed tomography (CT), revealed free gas in the abdominal cavity, leading to the misdiagnosis. However, intraoperation exploration identified no perforation. Postoperative abdominal enhanced CT and multidisciplinary consultation confirmed a splenic abscess. The patient was successfully treated with ultrasound-guided percutaneous drainage and meropenem antibiotic therapy, resulting in complete recovery.

## Introduction

Splenic abscess is a rare infectious disease, with an incidence of approximately 0.05% to 0.7% in autopsy series [1, 2]. Its occurrence is often associated with risk factors, such as immunodeficiency diseases, diabetes, infectious endocarditis, splenic infarction and splenectomy, malignancies, hematological conditions [3–5]. Gastrointestinal perforation is an acute abdomen characterized by sudden onset of severe abdominal pain and secondary peritonitis, typically presenting with subdiaphragmatic free gas on imaging. This report describes a case of ruptured spleen abscess causing intra-abdominal free gas, which was misdiagnosed as a gastrointestinal perforation.

## Case report

**Figure 1:**
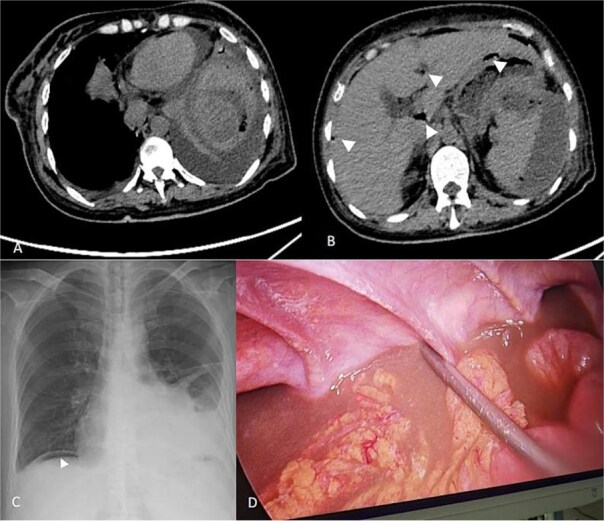
CT scan of spleen abscess (A) and free gas in the abdominal cavity (B) before the surgery and splenic Puncture. Free gas in the abdominal cavity on the chest X-ray (C). The scene during laparoscopic examination (D). Note: The triangle indicates the free gas in the abdominal cavity.

A 53-year-old female was admitted on 6 September 2025 with a 4-hour history of sudden abdominal pain. The pain originated in the epigastrium and was persistent, and rapidly generalized throughout the abdomen, accompanied nausea and vomiting. In order to seek treatment, the patient went to the emergency department of the People’s Hospital of Linxia Hui Autonomous Prefecture. Abdominal computed tomography (CT) demonstrated free gas beneath the diaphragm and around the liver, which mostly suggested digestive tract perforation or rupture; an abnormal splenic contour with perisplenic effusion; and signs of peritonitis with abdominopelvic fluid (Fig. 1A and B). Chest X-ray showed a digestive tract perforation combined with bilateral pleural effusion (Fig. 1C). Laboratory findings included white blood cells 15.95 × 10^9^ L, neutrophil ratio 90.30%, Albumin 26.6 g/L, procalcitonin 2.350 ng/ml. Physical examination revealed diffuse abdominal rigidity, tenderness, rebound tenderness, and diminished bowel sounds. The patient has a 10-year history of diabetes managed with acarbose, repaglinide, and subcutaneous insulin glargine (24 units).

The patient underwent emergency laparoscopic examination under general anesthesia on 6 September 2025. Intraoperatively, purulent exudate was adherent to the liver margin, gastric antrum, and small bowel, with copious pus and bloody fluid in the left upper quadrant and pelvis (Fig. 1D). After careful examination, no obvious perforation was found. The attachment of the splenic flexure was severe, making it prone to bleeding and unable to be separated. The spleen was not clearly visualized. The abdominal cavity was irrigated with warm saline, and drainage tubes were placed in the abdomen and pelvis. Postoperatively, the patient was transferred to the ICU due to critical condition. Meropenem was administered for infection control, alongside nutritional support, but clinical improvement was not observed.

**Figure 2:**
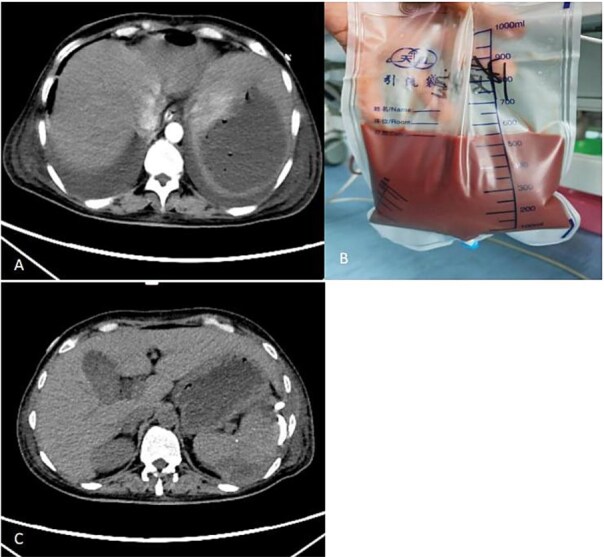
Enhanced CT scan after laparoscopic examination (A). Image of the drainage fluid obtained after spleen puncture (B). CT scan taken 6 days after the puncture and drainage of the spleen abscess (C).

On 8 September 2025, abdominal enhanced CT suggested a splenic abscess (Fig. 2A). After a multi-disciplinary discussion, the disease was finally diagnosed as splenic abscess. Ultrasound-guided percutaneous drainage of the splenic abscess was performed on 9 September 2025, yielding approximately 550 ml of pus (Fig. 2B). According to the drug sensitivity results, meropenem was continued to be administered intravenously, and metronidazole was used to flush the pus cavity through the drainage tube. The patient’s symptoms gradually resolved, with significant reduction in infection markers. Follow-up CT on 15 September 2025 showed marked shrinkage of the abscess cavity and minimal drainage output (Fig. 2C), prompting removal of the drainage tube.

## Discussion

Splenic abscess often presents with fever, leukocytosis, and left upper quadrant pain, and may also present with chest pain, pleural effusion, left shoulder pain, and abdominal distension [6, 7]. If the condition progresses, it can lead to septic shock. Treatment options include splenectomy for small or diffuse abscesses [8]; or image-guided percutaneous drainage combined with broad-spectrum antibiotics for large, localized abscesses [9, 10].

The onset of this patient was sudden, the symptoms were severe, the signs of peritoneal irritation were obvious, and free gas was seen in the abdominal film and abdominal CT, so it was misdiagnosed as gastrointestinal perforation. However, intraoperative findings excluded perforation. Moreover, the patient had severe adhesions in the left upper abdomen, which could easily lead to bleeding and intestinal wall damage, thus preventing a more detailed exploration. After ultrasound-guided puncture and drainage, the symptoms improved rapidly, confirming the splenic abscess diagnosis.

This case underscores a critical lesson: free intra-abdominal gas with abdominal tenderness combined with abdominal tenderness does not necessarily indicate a digestive tract perforation. Clinicians should conduct comprehensive history-taking, physical examination, and imaging review. Differential diagnoses must include splenic abscess rupture, particularly in patients with risk factors such as diabetes.
